# On the Influence of a Dilatant Asperity Patch on the Seismic Moment

**DOI:** 10.1007/s10659-025-10135-7

**Published:** 2025-05-21

**Authors:** P. A. Selvadurai, A. P. S. Selvadurai

**Affiliations:** 1 Swiss Seismological Service, Department of Earth and Planetary Sciences, ETH Zurich, Zurich, Switzerland; 2 Department of Civil Engineering, McGill University, Montréal, QC Canada H3A 0C3

**Keywords:** Embedded dilatant asperity, Dislocation theory, Seismic moment, Earthquake mechanics, 74A20, 74B05, 35J30, 45B05

## Abstract

This paper proposes a novel procedure to examine the influences of friction, dilatancy, and normal stresses at fault zones on the estimation of seismic moment. For illustrative purposes, the study focuses on a circular frictional dilatant patch located within a frictionless pre-compressed fault zone undergoing relative shear. When dilatancy occurs, the interface beyond the dilatant region may experience separation due to the normal stresses acting on the fault plane, affecting the deformational response of the pre-stressed asperity. This approach allows for an evaluation of the normal stress on the dilatant region, leading to a re-interpretation of the conventional definition of seismic moment. We compare our model against a comprehensive catalog of earthquakes spanning 16 orders of magnitude, utilizing seismologically inferred source properties as well as data from two separate experimental studies that directly measure the shear-dilatant response of shear fractures in both laboratory and field settings. Our findings indicate that friction-induced dilatancy exerts minimal influence on the estimation of seismic moment. However, we emphasize that the discrepancies between our direct measurements and inferred estimates of seismic moment highlight the need for focused campaigns and in situ and on-fault assessments of earthquake mechanics. **Plain Language summary**. The conventional definition of the *Seismic Moment* has been central to unifying information from plate tectonics, geology, geodesy, and seismology. It looks at how the ground moves along a fault plane and the strength of the surrounding rocks. However, it often overlooks other factors that might affect this movement, such as the stress on the fault and the local topography that can induce additional physical responses. This study explores how these additional factors, particularly a process called dilatancy, can change our understanding of the seismic moment. The authors found that while these factors do play a role, they have a minimal impact on the traditional definition of seismic moment initially proposed by Aki in 1966.

## Introduction

Earthquakes present substantial scientific and societal challenges, primarily due to the complex interactions occurring in fault zones at tectonic boundaries (e.g., Chester et al. [27]; Faulkner et al. [36]; Bürgmann [24]). Furthermore, human activities aimed at extracting geo-resources—such as fluid or gas extraction and subsurface injection—can inadvertently produce significant induced seismicity (Grigoli et al. [40]; Moein et al. [68]). Understanding fault processes is inherently challenging due to the wide range of spatiotemporal scales involved (Ben-Zion [15]). Moreover, direct observations of on-fault processes remain scarce, whether in natural tectonic environments or in dedicated field test sites (Zoback et al. [116]; Guglielmi et al. [42]; Villiger et al. [110]; Viswanathan et al. [111]; Obermann et al. [72]), thereby further complicating our comprehension of these phenomena.

For both natural and induced earthquakes, the physical mechanisms controlling them remain poorly understood due to complex and coupled hydromechanical and geochemical processes. One such process is fault dilatancy, whereby a discontinuity in a geomaterial can experience relative shear movement while allowing for dilation. This behavior can be largely influenced by the surface topography of the discontinuity (Bandis et al. [11]; Barton et al. [13]), frictional multi-contact mechanics (Bowden and Tabor [18]; Johnson [52]; Selvadurai and Glaser [85, 86]), the constitution of infill material hosted within the discontinuity (Niemeijer and Spiers [71]), and interactions with the parent material hosting the discontinuity due to incompatible movements at the contacting planes (Davis and Salt [32]; Selvadurai and Yu [98]; Tatone and Grasselli [105]) among others. Moreover, fault dilation is heavily linked to hydraulic properties and permeability evolution within the discontinuity, which are associated with changes in the fracture aperture (Brown [21]; Selvadurai and Nguyen [95]; Renshaw [77]; Boulon et al. [17]; Nguyen and Selvadurai [70]; Rezaei Niya and Selvadurai [79]; Huo et al. [48]).

Fault dilation with slip is intrinsically linked to the presence or absence of mechanical instabilities that can lead to earthquakes or produce aseismic transients (e.g., Marone [60]; Niemeijer and Spiers [71]). Various mechanisms have been identified through theoretical and laboratory studies; for instance, dilatancy can mitigate instability by decreasing the effects of fluid pressurization while enhancing permeability (Lockner and Byerlee [57]; Segall and Rice [87]; Scuderi et al. [85]; Brantut [20]; Proctor et al. [76]; Chen and Spiers [26]; Dal Zilio et al. [31]; Dunham [34]). Additionally, it can control frictional strength, which affects energy partitioning (Marone et al. [61]; Beeler et al. [14]; Scuderi et al. [86]), and induce dilatant hardening in regions of high fluid pressure (Frank [37]; Rice [80]; Segall et al. [88]; Scuderi and Colletini [84]; Agliardi et al. [3]). The coupling between dilation and fault zone permeability also impacts the critical nucleation size—a theoretical length scale that describes the minimum size an aseismically creeping patch must reach for slip to become unstable—as it is inversely proportional to the effective normal stress [e.g., Segall et al. [88]]. Clearly, the hydromechanical coupling associated with dilatancy during slip in discontinuities plays a critical role in various aspects of earthquake processes.

In this study, we step back and assume a patch (asperity) exists and is pre-compressed along an interface between two elastic bodies. When the patch is sheared, it experiences dilation following Selvadurai et al. [99]. A basic approach is taken to incorporate the effects of dilatancy, and we examine the scalar work produced during deformation. This methodology allows for a comparison with seismic moment—the dynamic equivalent of an area of the fault surface undergoing a dislocation in slip (Aki [4]) or strain (Burridge and Knopoff [25]) linked through the material rigidity. The seismic moment has been central to unifying information from plate tectonics, geology, geodesy, and seismology. Improved estimates for seismic moment that incorporate well-known physical mechanisms may help constrain the scaling behaviors of earthquake source parameters inferred from observations.

## Theoretical Background

**Fig. 1:**
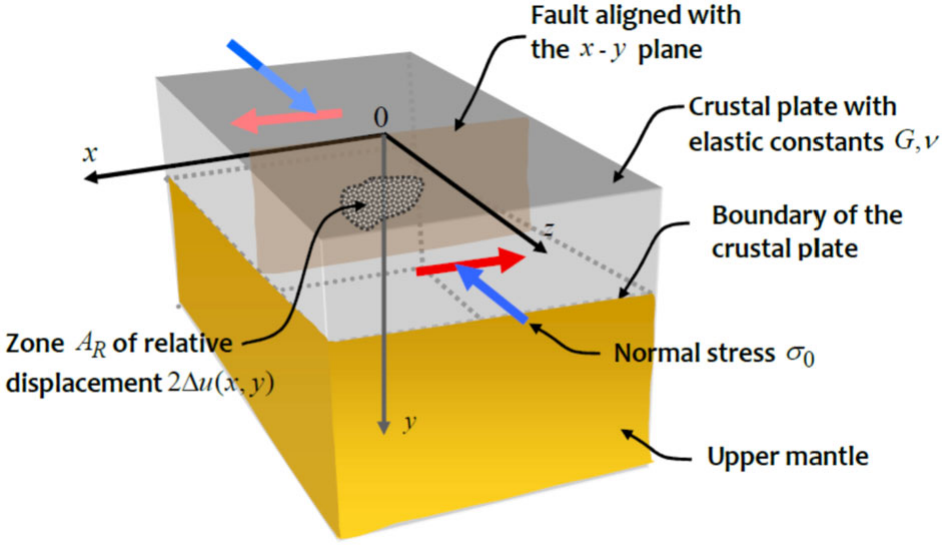
A slip dislocation, with relative displacements ($2\Delta u$), applied to a geometric discontinuity, with area $A_{R}$, used to represent the motions produced by a sudden earthquake in the Earth’s interior

Seminal studies have suggested that addressing the issue of seismic radiation released during sudden earthquakes within the Earth’s interior is likely related to solving a ‘dislocation’ problem (e.g., Knopoff and Gilbert [55]; Burridge and Knopoff [25]; Steketee [104]; Aki [4]). The origins of this problem are rooted in the mathematical theory of elasticity, tracing back to the works of Italian mathematicians (Volterra [112]; Somigliana [102]). As Steketee [104] noted, the original term ‘*distorsioni*’ was a translated form of ‘*dislocation*’ introduced by Love [58]. The problem revolves around the application of dynamic dislocation theory, which gives the elastodynamic radiation resulting from impulsive occurrence of an earthquake (e.g. Aki and Richards [6]). In the dislocation theory, the fault is viewed as a geometric discontinuity in which a discontinuous change in one component of the strain tensor or one component of the displacement vector is suddenly realized. The concept of the *seismic moment* introduced by Aki (equation (12) in [4]) (and further discussed by Brune [22]; Wyss and Brune [113]) quantitatively relates the moment obtained from seismic waves to the area of fault surface and the amount of dislocation. Referring to Fig. 1, for a slip dislocation with non-uniform relative movement $2\Delta u(x,y)$, the seismic moment $M_{0}$ can be expressed in the form:

1 $$ M_{0} = \iint _{A_{R}} 2G(x,y) \Delta u(x,y) dxdy, $$

where $G(x,y)$ is the variation in the shear modulus of the medium in the immediate vicinity of the source (Burridge and Knopoff [25]) and noting that the slip dislocation is unidirectional and occurs over a zero thickness planar patch of size $A_{R}$. In the special case when the relative movement and the shear modulus exhibit no variations within the region $A_{R}$, the definition (1) reduces to the elementary form

2 $$ M_{0} = GA_{R}2\Delta u, $$

where the relative motion $2\Delta u$, is oriented in the $x$-direction in Fig. 1, and is equivalent to the average slip $\bar{u}$ in equation 12 of Aki [4].

It is evident that this conventional definition of the seismic moment does not account for frictional phenomena and dilatancy effects in the contact zone $A_{R}$. The objective of this paper is to examine the possible influences of these factors on the estimation of the seismic moment. The paper examines the idealized problem of a smoothly compressed geological interface, which contains a circular contact region that can experience (i) frictional forces during relative shear, (ii) uniform dilatancy in the shear zone, (iii) separation beyond the dilatant zone and (iv) degradation of dilatancy with increasing relative shear displacement. The mathematical analysis of the resulting unilateral contact problem is used to evaluate the dilatancy-induced normal force at the dilatant contact zone. We find that the normal force has a negative work component, which is incorporated into the definition of the seismic moment.

## Modelling of the Dilatancy-Induced Contact Processes

**Fig. 2:**
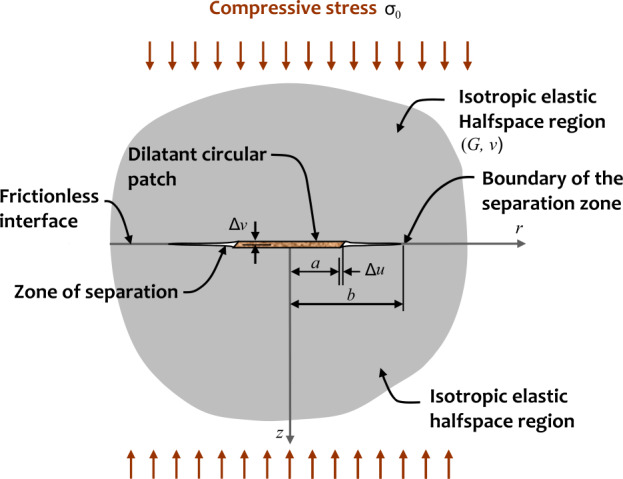
Indentation of the half-space regions by the dilatant displacements induced during shearing of the circular patch (see also Selvadurai et al. [99])

As described in Sect. 1, dilatancy is a well-studied and complex process characterized by the mechanics of discontinuities (e.g. Selvadurai and Yu [98]). In Sect. 2, we outline a more straightforward yet effective approach that employs elasticity and dynamic dislocation modeling to estimate the seismic moment (2). Additionally, we address the problem of a frictionless planar discontinuity; however, we apply a uniform normal stress, $\sigma _{0}$, to pre-compress this interface (Fig. 2). The interface contains a circular patch of radius $a$ that exhibits both friction and dilatancy, with both processes being activated only during relative shear at the interface. We assume that the geologic regions experience a relative movement $2\Delta u$ and in the process induces (i) a uniform dilatational displacement $2\Delta v$ in the contact zone, (ii) separation beyond the contact zone and (iii) re-establishes contact at the interface at the termination of the separation zone (Fig. 2). The region of separation will depend on the magnitude of the dilatant displacement ($2\Delta v$), the dimension of the circular region undergoing dilatancy ($a$), the elasticity properties of the surrounding geologic medium ($G$, $\nu $), and the normal stress($\sigma _{0}$)acting on the geologic media in contact. Due to the asymmetric nature of the shearing deformation, the region of separation beyond the dilation zone will have a shape that is non-symmetric about the $z $-axis. The resulting unilateral contact problem is a complex mathematical problem; even when the circular region that initiates the dilatancy effects at the contact zone is considered, an iterative technique needs to be adopted to locate the unknown boundary of the separation zone. In our approach, we assume that the location of the boundary of separation can be approximated by an axisymmetric circular line of radius $b$. This renders the analytical treatment of the contact problem shown in Fig. 2 tractable.

The procedure for examining the resulting unilateral contact problem requires the application of solutions to two elasticity problems involving the following *axisymmetric mixed boundary value problems*: (i) The first problem deals with the internal pressurization of an annular crack of internal radius $a$ and external radius $b$ by a uniform compressive stress $\sigma _{0}$ and (ii) the second involves the axisymmetric internal indentation of a penny-shaped crack of radius $b$ by a smooth rigid disc inclusion of radius $a$ and thickness $2\Delta v$. There are no known exact solutions for the two axisymmetric mixed boundary value problems posed above. Selvadurai and Singh [96, 97] and Selvadurai [90] developed approximate solutions to the governing integral equations by considering expansion for the unknown functions in the integral equations in terms of the ratio $a/b ( < 1)$. For detailed treatment of this problem the reader is urged to consult Selvadurai et al. [99]. This procedure also allows the calculation of the Mode I Stress Intensity Factor at the location $r = b$ for both the pressurized annular crack of external radius $b$, $K_{I}^{\sigma _{0}}$, and the Mode I Stress Intensity Factor $K_{I}^{\Delta v}$ at the boundary of the internally indented penny-shaped crack of radius $b$. The unknown radius of the zone of separation can be determined by imposing the condition that at the point of separation between regions of frictionless contact, the contact stress $\sigma _{zz}(r,0)$ vanishes as $r \to b$. This is equivalent to the vanishing of the combined stress intensity factor at the boundary: i.e.

3 $$ K_{I}^{\sigma _{0}} + K_{I}^{\Delta v} = 0 $$

**Fig. 3:**
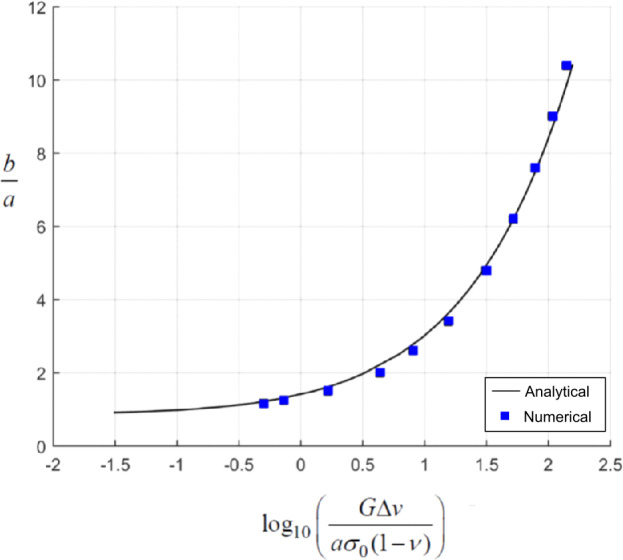
The influence of the pre-compression stress $\sigma _{0}$ and the dilatancy induced displacement $\Delta v$ on the radius of the zone of separation. [Solid line corresponds to the analytical estimate and the squares correspond to the computational results]

The result (3) gives rise to a characteristic equation of the form

4 $$ \left ( \frac{G\Delta v}{2\sigma _{0}a(1 - \nu )} \right )cF_{\Delta v}(c) - F_{\sigma _{0}}(c) = 0 $$

which can be solved to determine the non-dimensional location of the separation boundary $c = a/b$ where the functions $F_{\Delta v}(c)$ and $F_{\sigma _{0}}(c)$ are given in the Appendix. It is noted that the radius of the zone of separation depends on the non-dimensional parameter $G\Delta v/a\sigma _{0}(1 - \nu )$. Since the expressions for $F_{\Delta v}(c)$ and $F_{\sigma _{0}}(c)$ are approximate power series expansions in terms of $c$, the accuracy of the procedure for determining the separation radius was investigated by comparison with the results of the identical problem obtained through a finite element-based computational approach. The results of the comparison are shown in Fig. 3 and provides an adequate proof of the validity of the mathematical approach based on the series approximation solution of the governing integral equations.

The results from the solution of the two mixed boundary value problems described previously can also be used to develop an expression for the resultant normal force $P_{N}$ induced in the contact region due to the dilatancy-induced normal indentation $\Delta v$. The expression for the normal force can be given in the form

5 $$ P_{N} = \sigma _{0}\pi a^{2} + \frac{4aG\Delta v}{(1 - \nu )}P_{N}^{\Delta v}(c) - \sigma _{0}\pi a^{2}P_{N}^{ \sigma _{0}}(c) $$

where $P_{N}^{\Delta v}(c)$ and $P_{N}^{ \sigma _{0}}(c)$ are presented in the Appendix. In the limit when $\Delta v \to 0$, the total force exerted on the contact zone should reduce to $\sigma _{0}\pi a^{2}$. It is evident that there is a residual correction that occurs as a result of the power series approximation solution. In the limit when $c \equiv 1$, we obtain $P_{N}^{ \sigma _{0}}(1) \approx 0.0272$ and additional terms in the power series for $P_{N}^{ \sigma _{0}}(c)$ should be included to ensure that $P_{N}^{ \sigma _{0}}(c) \to 0$ as $c \to 1$. For the purposes of the ensuing developments we shall assume that the error of approximately 3% can be neglected and the expression for $P_{N}^{ \sigma _{0}}(c)$ can be retained for consistency but can be neglected in any calculations.

## Relationship Between the Shear and Dilatancy Displacements

The shearing movement at frictionally locked, and precompressed asperity induces displacement normal to the direction of shearing displacement. These observations trace back to early studies by Reynolds [78], Mead [65], and others, highlighting volume expansion in dense granular media subjected to shear. Recently, the concept of dilatancy has been used to explore the mechanics of contact between surfaces that undergo expansion due to surface irregularities in conforming and non-conforming interfaces. Key developments in this area are referenced by Taylor [106], Brace et al. [19], Frank [37], Jaeger [51], Michałowski and Mroz [66], Segall and Rice [87], Plesha [75], Selvadurai and Boulon [92], Selvadurai and Nguyen [95], Davis and Selvadurai [33], and Selvadurai and Yu [98]. Various relationships between shear and dilatancy have been proposed (e.g. Zhao and Cai [115]; Bianchi et al. [16]); however, a simpler formulation is adopted here.

To incorporate the effects of dilatancy, the basic approach proposed by Taylor [106] assumes a linear relationship between the shear displacement ($\Delta u$)on a dilatant interface and the dilatant displacement ($\Delta v$), expressed as:

6 $$ \Delta v = C_{T}\Delta u\tan \alpha , $$

where $\alpha $ is the dilatancy angle and $C_{T}$ is an non-dimensional arbitrary constant.

The linear dependency of dilatant displacement on shear displacement imposes specific restrictions: (i) dilatancy processes should exhibit symmetry with respect to the relative shear displacement, and (ii) they should incorporate micro-mechanical processes that may cause degradation of the dilatancy angle ($\alpha _{0}$) as the relative shear displacement increases. To meet these requirements, the modified Taylor relationship can be expressed as:

7 $$ \Delta v = C_{S}\left ( \Delta u \right )^{2}\tan \alpha , $$

where $C_{s}$ is an arbitrary constant with units $L^{-1}$. For both models in (6) and (7), the degradation of the dilation angle as a function of shear displacement is given as:

8 $$ \tan \alpha = \exp \left ( - \lambda \left | \Delta u \right | \right )\tan \alpha _{0}, $$

where $\lambda $ is an arbitrary constant with units $L^{-1}$.

In the linear model (Eq. (6)), the dilatancy at the interface is independent of the normal stress acting on the discontinuity or the local normal stiffness. While this assumption may be valid for shallow discontinuities, it is likely insufficient for fractures located at significant depths in seismogenic regions of the crust. At these depths, the breakage of asperities and the evolution of the dilation angle (as described in Eq. (8)) must be considered.

A comprehensive approach to this problem incorporates factors such as the normal stress state, asperity profile and scale, elasticity, and the failure characteristics of the rock material at the interface. This approach provides a more accurate representation of dilatancy behavior under varying conditions. However, it requires significant experimental work to validate and refine these relationships (e.g. Bandis et al. [10]; Plesha [75]; Nguyen and Selvadurai [70]).

**Fig. 4:**
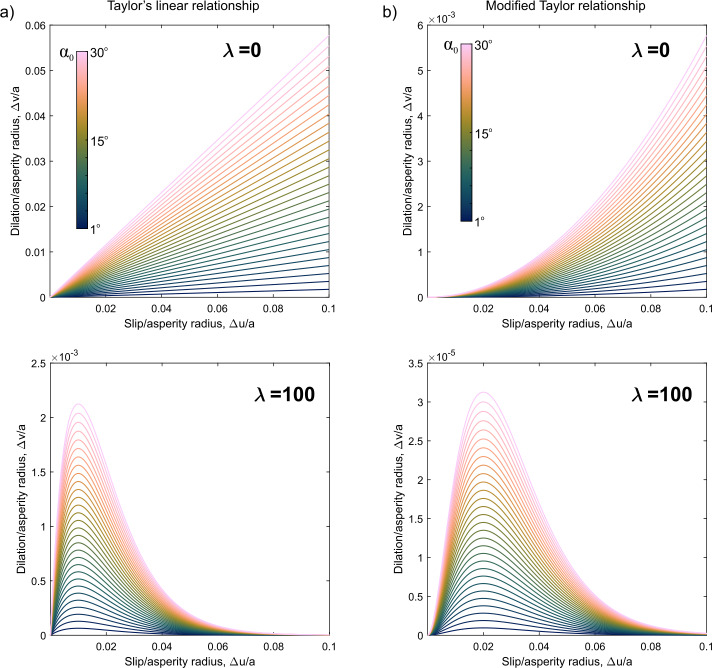
Shear-induced dilation models. (a) Taylor linear relationship described in Eq. (6) and (b) a modified-Taylor relationship described in Eq. (7) (see Selvadurai et al. [99]). The top and bottom panels show the response for degradation coefficients of $\lambda = 0$ and 100, respectively, and $C_{s} = 1\text{ mm}^{-1}$ and $C_{T} = 1$. The axes are normalized by the asperity radius $a$ and the shear displacements ($\Delta u$) was 10% of this value

In Fig. 4, we analyze the behavior of two shear-induced dilation models represented by Eqs. (6), (7), and (8). Figure 4a focuses on the linear model, specifically Eqs. (6) and (8), for $\lambda = 0$ (top panel) and $\lambda = 100$ (bottom panel). Meanwhile, Fig. 4b evaluates the modified Taylor relationship, encapsulated in Eqs. (7) and (8), also for $\lambda = 0$ (top panel) and $\lambda = 100$ (bottom panel).

In both cases, we assume that the total slip is 10% of the overall asperity radius ($a$). Additionally, we vary the dilation angle ($\alpha _{0}$) between 1^o^ and 30^o^, with the different angles indicated by the color of the curves. This analysis allows us to observe how variations in the dilatancy angles influences the behavior of the shear-induced dilation models.

**Fig. 5:**
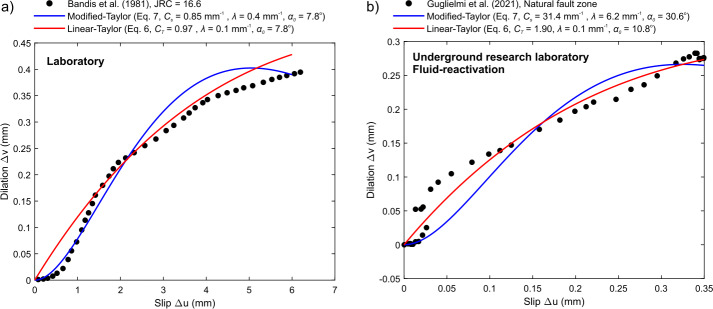
Fitting results describing the shear-dilation kinematic response in (a) a laboratory test from Bandis et al. [10] and (b) fluid-reactivation test in the Sanford Underground Research Facility (SURF) reported using an on-fault SIMFIP probe (Guglielmi et al. [43])

Despite its simplistic representation of shear-induced dilatancy, this model successfully captures behavior observed in laboratory experiments (Selvadurai et al. [99]). In Fig. 5a, we present results from an experiment conducted by Bandis et al. [10], in which direct shear testing was performed on a large natural fracture, measuring 300 mm in length, within weathered granite subjected to a normal stress of 0.96 MPa. This specific experiment focused on a rough surface characterized by a Joint Roughness Coefficient (JRC) of 16.6 (Barton and Choubey [12]). The data shown was digitized using *automeris.io*, a WebPlotDigitizer tool that utilizes computer vision to extract numerical data from various types of data visualizations.

Both the linear model (red curve, R$^{2} = 0.98$) and the modified Taylor relationship (blue curve, R$^{2} = 0.98$) were fitted to the experimental data through a least-squares regression approach, aiming to minimize the sum of the squares of the residuals. The results indicate that both models perform reasonably well in fitting the data. However, it is important to note that for these fits, the initial dilatancy angle ($\alpha _{0}$) was fixed at 7.8°. When allowing the regression to estimate the dilatancy angle ($\alpha _{0}$) simultaneously, we found that while the quality of the fits remained similar, the estimated values for the initial dilatancy angle dropped to approximately 2.5° for the linear model and 2.7° for the modified-Taylor relationship.

Figure 5b examines a second dataset at a different scale, focusing on measurements of opening and shear displacement obtained using a double-packer probe equipped with a high-resolution three-dimensional displacement borehole sensor known as the Step-Rate Injection Method for Fracture In-Situ Properties (SIMFIP) probe (Guglielmi et al. [41]). This experiment was conducted at an approximate depth of ∼1490 m in fractured metamorphic rocks at the Sanford Underground Research Facility (SURF) in South Dakota, USA (Kneafsey et al. [54]).

Hydraulic fluids were initially injected into a perforated section of the rock mass. The data presented here were digitally collected from Guglielmi et al. [43] using a similar approach as previously described. The first phase of this experiment involved hydraulic fracturing of the perforated section of the rock mass. Continuous fluid injection subsequently led to the growth of the newly formed fracture, resulting in the reactivation and hydraulic shearing of a preexisting discontinuity. The SIMFIP probe recorded the shear and normal displacements, which are illustrated in Fig. 5b.

Both the linear model (red curve, R$^{2}= 0.97$) and the modified Taylor relationship (blue curve, R$^{2} = 0.94$) were fitted to the experimental data using a least-squares regression algorithm. The results indicate that the linear model adequately represents the data once the fracture has mobilized. However, the initial onset of this mobilization may be somewhat skewed due to the initial phase of hydraulic fracturing, which could account for the less favorable fit of the modified Taylor model. This in situ behavior is complex and not fully understood; further efforts are needed to better measure real fault motions using on-fault probes like the SIMFIP. The framework described in this study allows for the integration of other shear-dilatancy constitutive laws, although the solutions and inferences for these alternative models may differ. More discussion is offered in Sect. 6.

We further modify (7) and (8) following Selvadurai et al. [99] to normalize the modified-Taylor shear-dilation relationship by the asperity radius ($a$). This follows:

9 $$ \Delta v = a\left ( \frac{\Delta u}{a} \right )^{2}\tan \alpha $$

where the dilation degredation angle becomes

10 $$ \tan \alpha = \exp \left ( - \lambda \left | \frac{\Delta u}{a} \right | \right )\tan \alpha _{0}. $$

With these kinematics of the shear deformation and dilatancy, the characteristic Eq. (4) for determining the radius of the boundary of separation can be represented in terms of $\Delta u$ in the form

11 $$ \left ( \frac{G}{\sigma _{0}(1 - \nu )} \right )\left ( \frac{\Delta u}{a} \right )^{2}\exp \left ( - \lambda \left | \frac{\Delta u}{a} \right | \right )\tan \alpha _{0} cF_{\Delta v}(c) - 2F_{\sigma _{0}}(c) = 0. $$

## Influence of Dilatancy on the Seismic Moment

The calculation of the seismic moment $M_{0}$ defined by Aki [4] is an estimate that represents the dynamic equivalence of a double couple and a slip dislocation. These are linked through the rigidity (i.e. the shear modulus of the medium) and the area of the fault surface undergoing the slip dislocation. The result (2) can be identified as a “scalar work” estimate of the dislocation movement. We retain the estimate of Aki [4] (see also Maruyama [63]) and include the influences of dilatancy at the frictional patch by considering the “scalar work” contribution of forces induced by the dilatant action $2\Delta v$ during the dislocational movement $2\Delta u$. The forces $P_{N}$ induced by dilatancy effects do negative work on the displacement $2\Delta v$. Omitting details, it can be shown that when effects of dilatancy are taken into consideration the seismic moment can be expressed in the form

12 $$ M_{0}^{*} = 2\pi a^{2}G\Delta u\left [ \textstyle\begin{array}{l} 1 - \left ( \frac{\sigma _{0}}{G} \right )\left ( \frac{\Delta u}{a} \right )\tan \alpha _{0}\exp \left ( - \lambda \left | \frac{\Delta u}{a} \right | \right ) \times \\ \left \{ 1 + \frac{G}{\pi \sigma _{0}(1 - \nu )}\left ( \frac{\Delta u}{a} \right )^{2}\exp \left ( - \lambda \left | \frac{\Delta u}{a} \right | \right )P_{N}^{\Delta v} - P_{N}^{\sigma _{0}} \right \} \end{array}\displaystyle \right ] $$

If terms of order greater than unity in ($\Delta u/a$), and the truncation error term $P_{N}^{\sigma _{0}}$ in (10) are neglected, the seismic moment for a circular patch undergoing dilatant shear can be expressed in the simplified form

13 $$ M_{0}^{*} = 2\pi a^{2}G\Delta u\left [ 1 - \left ( \frac{\sigma _{0}}{G} \right )\left ( \frac{\Delta u}{a} \right )\tan \alpha _{0}\exp \left ( - \lambda \left | \frac{\Delta u}{a} \right | \right ) \right ] $$

which now takes into account (i) the normal stress acting on the plane of shear, (ii) the dilatancy angle $\alpha _{0}$ and (iii) the dilatancy degradation parameter $\lambda $. As with the estimate of Aki [5], the Coulomb friction at the circular patch does not enter into the classical definition of the seismic moment, and (13) reduces to Aki’s classical result when $\alpha _{0} = 0$.

## Discussion

The paper presents a rational analytical approach for examining the influence of dilatancy at a fault zone in interpreting the classical measure of the “Seismic Moment”. To develop an analytical approach, the theoretical model is restricted to the study of a circular frictional-dilatant patch in a smooth fault zone. The theoretical developments culminate in a simplified expression for the seismic moment that also accounts for the work of dilatant forces accompanying shear-induced uniform dilatational deformations in the circular region.

This study treats the problem under the assumption that the asperity undergoes uniform dilation. While the mechanisms controlling this dilation are complex, their intricacies are ultimately not treated in this study but the framework lends itself to incorporating more advanced models. It is important to acknowledge this limitation. Experimental evidence indicates that the assumption of uniform dilation is a simplification; for example, dilation can mobilize and change with shear deformation (Zhao and Cai [115]) or vary in space due to local properties, time-history of slip and the slip rate will vary to compensate for coupled physical behaviors (e.g., Segall et al. [88]; Chen and Spiers [26]; Heimisson et al. [47]; Dal Zilio et al. [31]; Dunham [34]).

**Fig. 6:**
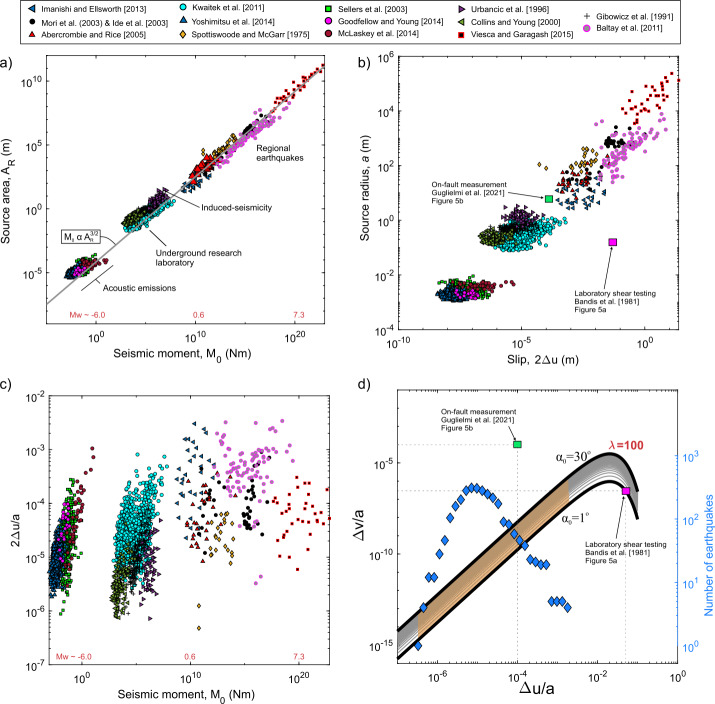
A composite of 14 studies of earthquake source properties taken from Selvadurai [91] with a total of 2796 earthquakes in the catalog. (a) Scaling relationship between source area and seismic moment. (b) Scaling relationship between slip and source radius (assuming shear modulus $G = 30$ GPa and a circular patch $A_{r} = \pi a^{2}$). (c) Scaling relationship between seismic moment and slip. (d) Shown is the normalized shear ($\Delta u$/$a$) and dilation ($\Delta v$/$a$) response determined from the modified-Taylor relationship (Eq. (8)) with $\alpha _{0}$ in [1^o^, 30^o^] and $\lambda =100$. Overlain is the frequency of earthquakes with respect to the $\Delta u/a$ determined for earthquake catalog (blue diamonds, referenced to the y-axis on the right-hand side). The orange region indicates expected levels of normalized dilation expected from the modified-Taylor relationship. Measurements from the laboratory and underground on-fault responses (see Fig. 5) are shown for reference in b) and d)

The composite analysis presented in Fig. 6 draws from 14 studies of seismic activity compiled by Selvadurai [91], encompassing a total of 2,796 earthquakes (Abercrombie and Rice [2]; Baltay et al., [8]; Collins and Young [30]; Gibowicz et al. [38]; Goodfellow and Young [39]; Ide and Beroza [49]; Kwiatek et al. [50]; McLaskey et al. [56]; Mori et al., [64]; Sellers et al. [89]; Spottiswoode and McGarr [103]; Urbanic et al. [108]; Viesca and Garagash [109]; Yoshimitsu et al. [114]). This dataset includes a broad range of seismic events, from laboratory-recorded acoustic emissions (M$_{\mathrm{w}} \sim -9$) to regional earthquakes (M$_{\mathrm{w}}\ \sim $7), where the moment magnitude $M_{w}$ can be related to Aki’s seismic moment following $M_{w} = (2/3)\log _{10}M_{0} - 6.03$ (Hanks and Kanamori [46]). Seismologically inferred estimates of the seismic moment $M_{0}$ and source radius $a$ were computed using spectral properites of the incoming wave phases (see details in Aki [5]; Brune [23]; Madariaga [59]; Hanks [45]; Aki and Richards [6]; Selvadurai [91]).

In Fig. 6a, we compare the source area $A_{R}$ to seismic moment $M_{0}$ under the assumption of a circular rupture patch. This observation is relevant to the scaling of the seismic source spectrum, following $M_{0} \propto A_{R}^{3/2}$, as discussed in previous studies (Aki [5]; Shi et al. [101]). Using estimates of source radius and seismic moment, and assuming circular fault patches, the slip $\left ( 2\Delta u \right )$ was computed using dislocation theory (as represented in Eq. (2)). A shear modulus $G = 30\text{ GPa}$, was assumed and Fig. 6b displays this scaling relationship. Added to Fig. 6b are estimates of slip and source size from the experimental studies in Fig. 5. We consider the maximum shear deformation of $\Delta u= 6.2\text{ mm}$ from the laboratory test (Fig. 5a) and $\Delta u= 0.35\text{ mm}$ from the field test (Fig. 5b). We assume the size of the patch undergoing shear and dilation is $a = 150\text{ mm}$ for the experimental fault half-length by Bandis et al. [10] and $a = 6\text{ m}$ as the reactivation fault half-length in the natural setting estimated by Guglielmi et al. [43].

Figure 6c compares the slip normalized by source radius ($2\Delta u/a$) to the seismic moment. No clear trend emerges, as the normalized slip exhibits moment-independent scaling behavior. This independence is expected because the normalized slip is proportional to the stress drop $\Delta \sigma $ of a penny-shaped crack subjected to uniform shear displacement (Eshelby [35]). Stress drop is given as

14 $$ \Delta \sigma = \frac{7M_{0}}{16a^{3}} = \frac{7\pi}{16}G\left ( \frac{2\Delta u}{a} \right ). $$

Previous studies have shown moment-independent scaling of stress drop (Cocco et al. [28]; Selvadurai [91]); however, this remains a topic of considerable debate (Shearer et al. [100]; Cocco et al. [29]). In this study, stress drop values range from 0.1 to 10 MPa (see Selvadurai [91]), comparable to estimates in larger global catalogs (Hanks [44]; Allmann and Shearer [7]). Some trends appear within individual studies reported here, particularly at smaller scales, but it remains unclear if these patterns result from stress interactions, correlations among source parameters, or study-dependent features related to sensor or array sensitivities (Cocco et al. [28, 29]).

Baltay et al. [9] conducted a recent community stress drop validation study focused on the 2019 Ridgecrest, California, earthquake sequence. In this study, researchers were invited to use a common dataset to independently estimate comparable measurements using various methods. They found that trade-offs between spectral estimates of stress drop and source radius can contribute to some of the observed dispersion and are influenced by methodological approaches. These discrepancies may have significant implications for understanding the complexity and variability of the source mechanics of seismic events (Abercrombie [1]).

In the validation study, Baltay et al. [9] present a comprehensive compendium of earthquakes, where in some cases the stress drop reaches as high as 1000 MPa (see Fig. 1 therein). While these events are notable outliers, they are consistent with shear failure observed in intact rocks in confined settings during laboratory tests (e.g., Ohnaka [73]), where fault dilation plays a significant role during the preparation process (Paterson and Wong [74], Salazar Vásquez et al. [82]). Given that earthquakes occur in sequences and produce complex spatial and temporal clustering (e.g., Mirzahi et al. [67]), it is essential to reduce uncertainty and improve our interpretation of potential outlier events to better understand the collective behavior of earthquakes (Ben-Zion [15]).

Figure 6d compares normalized slip ($2\Delta u/a$) from our earthquake catalog to estimates of dilation derived from our modified-Taylor dilation model (Eq. (7); see also Fig. 4b, with $\lambda = 100$ and $C_{S} = 1\text{ mm}^{-1}$). We plot the probability distribution function of the normalized slip, which ranges from $2\Delta u/a \in [5 \times 10^{-7}, 4 \times10^{-3}]$ with an arithmetic mean $6.4 \times 10^{-5}$. The region corresponding to these ranges of slip ratios is highlighted in orange. In our analysis, we find that for this kinematic shear-dilation model and for typical ranges of normalized slip interpreted from source models, the values of normalized dilation are notably small and will likely play little role in estimates of the seismic moment using (13) as discussed later.

For reference, we have included normalized shear and dilation measurements presented in Fig. 5 from Bandis et al. [10] and Guglielmi et al. [43] in Fig. 6b. Interestingly, the on-fault measurements reported by Guglielmi et al. [43, see also Figure 5b] fall within the range of estimates of normalized seismic slip, approximately $2\Delta u/a\sim 1 \times 10^{-4}$; however, the magnitude of normalized dilation ($2\Delta v/a\sim 1 \times 10^{-4}$) is significantly higher than predicted by our model for the chosen parameters. In contrast, the levels of normalized dilation for the laboratory tests conducted by Bandis et al. [10] are around $2\Delta v/a\sim 2 \times10^{-3}$, which aligns well with the model predictions. However, this is only achieved with correspondingly high levels of normalized slip in these laboratory tests, approximately $2\Delta u/a\ \sim 4 \times10^{-2}$, which far exceeds the estimates derived from the earthquake catalog. According to Eq. (14), this level of shear deformation is equivalent to an earthquake with $\Delta \sigma \sim1650\text{ MPa}$.

These observations highlight the discrepancies between field measurements and laboratory results, suggesting that different mechanisms may be at play in natural settings compared to controlled experiments in the laboratory. Further investigation into these differences could provide valuable insights into the complexities of shear-dilation behavior under varying conditions. Developing a better understanding on physical mechanisms controlling the shear-dilation model coefficients ($C_{T}$, $C_{S}$, $\alpha _{0}$, $\lambda $) through increased campaings targeting on-fault measurements of shear-dilatant response of natural geologic features would be a useful exercise.

Some factors could contribute to the discrepancy in Fig. 6. The fault patch in the experimental studies that was reactivated did not culminate in a macroscopic dynamic rupture, which could differ significantly from the estimates of source properties observed in the earthquake catalog. This suggests that the mechanics at play during slow reactivation of rough-rough faults may not be fully captured by broader scaling relationships of dynamic ruptures during an earthquake.

The preparation for earthquakes may occur over a more volumetric region, indicative of substantial dilative components (Kato and Ben-Zion [53]; Martínez-Garzón and Poli [62]; Salazar Vásquez et al. [82]). However, once a strain localizes (Rudnicki and Rice [81]), the ensuing accelerating rupture will localize and tends to propagate along preexisting principal slip zones that are geometrically thin, which could exhibit lower dilation during this phase of the earthquake, which in our model (Eq. (13)), plays less of a role.

For the case were fluid reactivation is used to produce an instability, we note that the mechanics governing fluid-reactivated may differ from those of induced by natural tectonic loading (Frank [37]; Rice [80]; Segall and Rice [87]; Segall et al. [88]; Scuderi et al. [85]; Brantut [20]; Proctor et al. [76]; Scuderi and Colletini [84]; Agliardi et al. [3]; Dal Zilio et al. [31]; Dunham [34]). Factors such as pore pressure changes, fluid migration, and the resulting stress conditions can significantly alter fault behavior, necessitating dedicated experiments at larger scales to fully resolve and understand these interactions. These considerations underscore the complexity of fault mechanics and highlight the need for further research to reconcile differences across various studies and contexts. Understanding these factors will be crucial for developing accurate predictive models for seismic behavior that take into account well-observed behaviors that may impact our theoretical estimates of earthquake source properties such as the seismic moment.

## Concluding Remarks

As an example, consider a circular dilatant patch of radius $a \approx 1 \text{ km}$ that is located at a fault plane situated at a depth of $D \approx 10 \text{ km}$, in a basaltic geologic medium with $G \approx \text{ 30 GPa}; \nu \approx 0.25$ and bulk density $\rho \approx 2700 \text{ kg}/\text{m}^{3}$. The dislocational shear movement is assumed to be $2\Delta u= 1\text{ m}$ and the peak dilatancy angle $\alpha _{0} \approx 10^{\mathrm{o}}$; we assume that the degradation of the dilatancy angle can be neglected by setting $\lambda = 0$. Using Eq. (2) we obtain an estimate of $M_{0} \approx 9.4 \times 10^{16}\text{ Nm}$, equivalent to a $M_{w}$ 5.3 earthqauke. For a fault oriented vertically in a strike-slip configuration (Fig. 1), the normal stress acting on the fault plane is $\sigma _{0} = \rho gD\nu /(1 - \nu ) \approx 113 \text{ MPa}$, where $g$ is the gravitational constant. Using this data in (13), the non-dimensional seismic moment $\bar{M}_{0}( = M_{0}^{*}/M_{0}) \approx 0.999999666$. The calculations clearly show that the effects of dilatancy are influenced, in addition to the dilatancy angle $\alpha $, by the product of the first and second non-dimensional parameter groups $(\sigma _{0}/G) < 1$ and $(\Delta u/a) < 1$, respectively. The product of the second non-dimensional parameter ($\Delta u/a$) is shown to range from 10^−6^ to 10^−1^ but on average is quite small $(\Delta u/a) \approx 6.4 \cdot 10^{ - 5}$. For most geologic media, the seismogenic depth of the dislocation zone and its dimensions leads to $(\sigma _{0}\Delta u/Ga)\tan \alpha _{0} \ll 1$, and it could be concluded that the measure of the seismic moment proposed by Aki [4] is expected to be valid for *both non-dilatant* and *dilatant* fault zones.

For fluid-reactivated faults, Terzaghi’s [107] principle of effective normal stress $\sigma _{eff}$ is known to reduce the compressive (normal) stress by counteracting the lithostatic load with pressurized pore fluids $P_{f}$ in the fault as $\sigma _{eff} = (\sigma _{0} - P_{f})$ (see Scholz [83]). We note that this will only further reduce the value first non-dimensional parameter ($\sigma _{0}/G$) since $\sigma _{eff} \le \sigma _{0}$. Morever, at depth where the compressive stress may produce larger ratios of the first product ($\sigma _{0}/G$), the temperature effects may limit the brittle response, reducing the dilative behavior in the material (Paterson and Wong [74]).

We note that there exists discrepancies between laboratory and *in situ* efforts to understand shear-induced dilation (Fig. 6). This highlights challenges in understanding how fault zones prepare and the shear-dilatancy response may vary across scales where geologic complexity increases (Fig. 5). Compaction and dilation within thin discontinuities may affect energy partinioning in more nuanced ways than described in this model. Increased efforts in field-scale campaigns targeting dedicated on-fault monitoring of shear and normal deformation, will enhance our understanding of earthquake mechanics and fault behavior beyond laboratory testing. Specifically, improving our kinematic understanding of shear-dilatancy during the preparatory phase and, in particular, during that passage of a significant earthquake rupture, would greatly benefit our knowledge of the mechanics in principal slip zones that host earthquakes.

## Data Availability

No datasets were generated or analysed during the current study.

## Appendix

The functions $F_{\Delta v}(c)$, $F_{\sigma _{0}}(c)$, $P_{N}^{\Delta v}$ and $P_{N}^{\sigma _{0}}$ referred to in the text are defined as follows:

$$ F_{\Delta v}(c) = \left [ \textstyle\begin{array}{l} \frac{4c}{\pi} + \frac{16c^{2}}{\pi ^{3}} + c^{3}\left ( \frac{64}{\pi ^{5}} + \frac{4}{3\pi} \right ) \\ + c^{4}\left ( \frac{80}{9\pi ^{3}} + \frac{256}{\pi ^{7}} \right ) + c^{5}\left ( \frac{448}{9\pi ^{5}} + \frac{1024}{\pi ^{9}} + \frac{4}{5\pi} \right ) \end{array}\displaystyle \right ], $$

$$ F_{\sigma _{0}}(c) = \left [ \textstyle\begin{array}{l} 1 - \frac{4c}{\pi ^{2}} - \frac{16c^{2}}{\pi ^{4}} - c^{3}\left ( \frac{1}{8} + \frac{64}{\pi ^{6}} \right ) \\ - c^{4}\left \{ \frac{16}{3\pi ^{4}} + \frac{4}{\pi ^{2}}\left ( \frac{1}{24} - \frac{8}{9\pi ^{2}} + \frac{64}{\pi ^{6}} + \frac{4}{9\pi ^{3}} \right ) \right \} \\ - c^{5}\left \{ \frac{16}{\pi ^{4}}\left ( \frac{1}{24} - \frac{8}{9\pi ^{3}} + \frac{64}{\pi ^{6}} + \frac{8}{9\pi ^{2}} \right ) + \frac{256}{9\pi ^{6}} - \frac{4}{15\pi ^{2}} \right \} + \mathrm{O}(c^{6}) \end{array}\displaystyle \right ], $$

$$ P_{N}^{\Delta v} = \left [ \textstyle\begin{array}{l} \left ( 1 + \frac{4c}{\pi} \right ) + \frac{16c^{2}}{\pi ^{4}} + c^{3}\left ( \frac{64}{\pi ^{6}} + \frac{16}{9\pi ^{4}} - \frac{8}{9\pi ^{2}} \right ) \\ + c^{4}\left ( \frac{256}{\pi ^{8}} + \frac{64}{9\pi ^{4}} \right ) + c^{5}\left ( \frac{10240}{\pi ^{10}} + \frac{9600}{225\pi ^{6}} + \frac{92}{225\pi ^{2}} \right ) \end{array}\displaystyle \right ], $$

$$ P_{N}^{\sigma _{0}} = \left ( \textstyle\begin{array}{l} - \frac{8}{\pi ^{2}c} + \left \{ 1 - \frac{32}{\pi ^{4}} \right \} + 8c\left \{ \frac{1}{\pi ^{2}} - \frac{48}{\pi ^{6}} \right \} \\ - \frac{c^{2}}{9\pi ^{8}}\left \{ 4608 + \pi ^{3}\left ( 32 - 64\pi + 3\pi ^{3} \right ) \right \} \\ - \frac{4c^{3}}{45\pi ^{10}}\left \{ 23040 + \pi ^{3}\left ( - 320 + 480\pi + 15\pi ^{3} + 6\pi ^{5} \right ) \right \} \\ - \frac{c^{4}}{675\pi ^{12}}\left \{ 5529600 + \pi ^{3}\left ( - 76800 + \pi \left [ \textstyle\begin{array}{l} 192000 \\ + \pi ^{2} ( 3600 \\+ \pi \{ - 320 + 3168\pi + 45\pi ^{3} \} ) \end{array}\displaystyle \right ] \right ) \right \} \end{array}\displaystyle \right ). $$
